# Bisphosphonate Treatment Alters the Skeletal Response to Mechanical Stimulation in Children With Osteogenesis Imperfecta: A Pilot Study

**DOI:** 10.1002/jbm4.10592

**Published:** 2022-03-01

**Authors:** Siva Sithambaran, Rachel Harrison, Sujatha Gopal‐Kothandapandi, Alan Rigby, Nick Bishop

**Affiliations:** ^1^ Department of Oncology and Metabolism University of Sheffield Sheffield UK; ^2^ Sheffield Children's NHS FT Sheffield UK; ^3^ University of Hull Hull UK; ^4^ Hull York Medical School Hull UK

**Keywords:** ANALYSIS/QUANTITATION OF BONE, ANTIRESORPTIVES, BIOCHEMICAL MARKERS OF BONE TURNOVER, CLINICAL TRIAL, OSTEOGENESIS IMPERFECTA

## Abstract

Children with osteogenesis imperfecta (OI) are commonly treated with bisphosphonates. We investigated the skeletal response to mechanical stimulation in children with OI before and after bisphosphonate treatment. Twelve children with OI, naïve to bisphosphonate treatment, stood on a high‐frequency (30 Hz), low‐amplitude (50 to 200 μ) vibrating platform (Marodyne LivMD) for 10 minutes daily (2.5 minutes × 4 with interspersed 1‐minute rest periods) for 7 days (whole body vibration [WBV] 1; day (D) 1–7), followed successively by 5 weeks' monitoring without intervention, 6 weeks' risedronate treatment, 1 week of WBV (WBV2; D85–91), and 1 week without intervention (D92–98). Procollagen type I N‐terminal propeptide (P1NP), bone‐specific alkaline phosphatase (BSALP), and carboxy‐terminal telopeptide of type I collagen cross‐link (CTX) were measured at baseline and intervals bracketing periods of vibration and risedronate treatment. Both P1NP and CTX rose to D8 (18.4%, 13.8%, *p* < 0.05, respectively), plateaued, then rose again at D43 (19.8%, 19.2%, respectively, *p* < 0.05 versus baseline). At D85 (after risedronate) both P1NP and CTX had fallen to pre‐WBV1 levels. A significantly smaller increase in P1NP was found after WBV2 (D85–91) at D92 (3.5%, 9.2%, respectively) and D99 versus after WBV1 (both *p* < 0.05). BSALP changed little after WBV1, fell during risedronate, and rose toward baseline after WBV2. We thus showed that WBV increased bone formation and resorption; that increase was attenuated after risedronate. The early increase in P1NP and CTX (D8) after WBV1 suggests increased osteoid formation within existing remodeling units but not increased mineralization. Later increases in P1NP/CTX (D42) suggest increased remodeling cycle initiation after WBV. Risedronate suppressed both biomarkers. The lower increase in P1NP/CTX after WBV2 suggests limited capacity to increase osteoid formation from existing “early stage” osteoblasts and a possible “hangover” effect of risedronate on remodeling activation. These results provide insights into both the response to WBV, ie, mechanical stimulation, and the effect of antiresorptive therapy in children with OI. © 2021 The Authors. *JBMR Plus* published by Wiley Periodicals LLC. on behalf of American Society for Bone and Mineral Research.

## 1 Introduction

Osteogenesis Imperfecta (OI) is a spectrum of genetic disorders with decreased bone mass and increased bone fragility. It is characterized by fractures associated with minimal or absent trauma, dentinogenesis imperfecta, blue sclerae, and progressive hearing loss. OI has a prevalence of approximately 11/100,000,^(1^ ^)^ and the fracture risk is greatest during childhood.^(^ ^2^ ^)^

At a tissue level, the characteristic feature of bone in OI is its brittle nature.^(^ ^3^ ^)^ This is largely because of an excess in the amount of mineral present relative to the fiber content of the bone material and also the altered 3‐dimensional structure of the fibrous component of the matrix. At a microscopic level, there is a reduced amount of bone tissue, with both the cortical and trabecular bone being affected. The cortices are narrower, with large intracortical pores, and trabecular connectivity is disrupted. The overall width of tubular long bones is reduced. This combination of material brittleness, disrupted microarchitecture and reduced bone size all combine to reduce fracture resistance and increase bone fragility. In children with OI, bone turnover is increased; the replacement of resorbed bone is defective compared with normal, and the density of osteocytes (terminally differentiated osteoblasts that are embedded within the bone substance and that form part of the pressure‐sensing network in bone) is increased.^(^ ^4^ ^)^

Bisphosphonates are potent antiresorptive agents that inhibit osteoclast function; the nitrogen‐containing bisphosphonates act on specific enzymes in the mevalonate pathway, reducing prenylation activity and effectively poisoning the osteoclasts, resulting in reduced function and apoptosis.^(^ ^5^ ^)^

Bisphosphonates have been widely used in the treatment of children with OI over the last 25 to 30 years. At a tissue level, the changes found in children with OI treated with bisphosphonates include an increase in trabecular number, thickness, and connectivity, reduced intracortical porosity, and increased cortical thickness.^(^ ^6^ ^)^ At a whole bone level, restoration of vertebral shape and size as well as increased bone width in long bones and reduced long bone bowing deformity have been reported.^(^ ^7^ ^)^ However, two recent reviews have highlighted that despite these observed changes, the effects of such therapy on fracture frequency are equivocal, though multiple studies (randomized controlled trials and observational cohorts) report this independently, and no studies report an increased fracture rate with treatment.^(^ ^8^, ^9^ ^)^

Whole body vibration (WBV) therapy is targeted at musculoskeletal strengthening and has been trialed in a variety of conditions. WBV has been shown to have therapeutic advantage in various osteopenic preclinical models and populations such as postmenopausal women (improved mobility, muscle strength, postural strength, and bone density) and children with osteogenesis imperfecta (improved mobility).^(^ ^10^, ^11^, ^12^, ^13^, ^14^, ^15^ ^)^

An alternative approach to the use of WBV is to regard it as a means to provide a standardized dose of mechanical stimulation to the skeleton. Our studies of WBV in healthy boys showed that a short period of exposure—5 days of 10 minutes WBV per day—could increase a bone formation marker in excess of one reflecting bone resorption.^(^ ^16^ ^)^ We have also used the same short period of WBV stimulation to demonstrate differences in the skeletal response of children whose mothers were enrolled in the MAVIDOS study and received vitamin D supplementation as opposed to placebo during pregnancy.^(^ ^17^ ^)^ The bone turnover markers assessed reflect type 1 collagen formation (procollagen type 1 N‐terminal propeptide), the mineralization of bone matrix (bone‐specific alkaline phosphatase) and type 1 collagen destruction (C‐terminal telopeptide of type 1 collagen).

It is unclear to what extent the skeleton in children with OI is responsive to mechanical stimulation; it may be that it is normally responsive, but the osteoblastic activity that occurs in response to mechanical stimulation is defective. It could be that the higher intrinsic levels of bone turnover in OI reduce the capacity of bone to further respond by increasing bone formation in excess of bone resorption. It might also be the case that the use of bisphosphonates alters the ability of bone tissue and cells to respond to mechanical stimulation. As bisphosphonates inhibit bone resorption, bone formation is also inhibited as a result of reduced remodeling; reduced osteoblast surface has been shown in the bone biopsies of OI children receiving bisphosphonates.^(^ ^6^ ^)^

We undertook this study to test the hypothesis that bisphosphonate treatment would reduce the response of the skeleton, as measured by changes in biomarkers reflecting bone formation and resorption, to whole body vibration in children with OI.

## 2 Materials and Methods

This single‐center interventional trial was approved by the South Sheffield Research Ethics Committee (ref 17/YH/0018) and registered with Clinicaltrials.gov, identifier NCT03208582. Participants attending the OI service at Sheffield Children's Hospital were approached regarding the study. The eligibility criteria were: ages 4 to 16 years; able to speak fluent English; diagnosed with osteogenesis imperfecta; able to stand; and naïve to treatment with bisphosphonates. Children with OI in our clinic are recommended to take a vitamin D supplement; those with a low calcium intake (based on reported low dairy product intake) are asked to take a calcium supplement. All the children in this study had previously received vitamin D; none had had additional calcium. Children were excluded if one or more of the following criteria were met: presence of other chronic illnesses including renal failure likely to affect bone metabolism; balance problems; recent fracture (in the last 6 months); recent (last 12 months) or current treatment likely to affect bone (excluding inhaled or intermittent oral therapy with steroids for asthma); involvement in another interventional research project; hypocalcemia; pregnancy or lactation; or known hypersensitivity to risedronate or any of the excipients.

Participants stood on a high‐frequency (30 Hz), low‐amplitude (50–200 μ) vibrating platform (Marodyne LivMD, BTT Health, Inning am Ammersee, Germany) for 10 minutes daily (2.5 minutes × 4 with interspersed 1‐minute rest periods) (WBV1) for 7 days (D1–7), followed successively by 5 weeks without further intervention, 6 weeks' oral risedronate treatment with accompanying calcium and vitamin D, 1 week WBV (WBV2, D85–91), and 1 further week without intervention (D92–98).

Risedronate, calcium, and vitamin D were given during the 6‐week period from D43 to D84.

The dose of risedronate administered (parentally supervised) was 1 mg/kg/week rounded to the nearest 5 mg using film‐coated tablets of 5 mg and 35 mg. Risedronate was administered first thing in the morning as per manufacturer's instructions in the fasting state with a large glass of water, with nothing further to eat or drink other than tap water for the next 30 minutes. Vitamin D and calcium were given daily in the evening as Calcichew 500 mg/200 IU tablets (Takeda Ltd, High Wycombe, UK), 1 tablet for participants weighing less than 30 kg and 2 tablets for participants weighing 30 kg or more. Risedronate and Calcichew tablets were prescribed through the Sheffield Children's Hospital pharmacy; any unused medication was returned and checked in order to ascertain compliance.

Blood samples were taken at baseline on D1, and on D8, D15, D43, D85, D92, and D99, bracketing the periods of vibration and drug treatment. All samples were taken in the fasting state before 9:00 a.m. Samples were spun at 2500 rpm for 10 minutes at 4°C and centrifuged samples stored at −80°C. Samples were analyzed in a single batch for bone‐specific alkaline phosphatase (BSALP), procollagen type 1 N‐terminal propeptide (P1NP), and C‐terminal telopeptide of type 1 collagen (CTX) in the Bone Biochemistry Laboratory, University of Sheffield, using an Elecsys Cobas E411 automated immunoassay system interassay coefficients of variation (CVs) 2.8% to 8.4% for CTX and <1.7% for P1NP. BSALP was measured on an iSYS automated immunoassay system (Immunodiagnostics Ltd., Boldon, UK) with interassay CV 4.2%.

Pubertal status of every patient was assessed using a sex‐appropriate pictorial scale. Pregnancy testing was carried out in the Sheffield Children's Hospital Clinical Research Facility before risedronate was given to any female subject aged 10 years or older.

Our choice of sample size is pragmatic and not informed by power. A literature review of sample size for pilot and feasibility studies found a few articles with recommendations for two‐arm trials but nothing of good quality for single‐arm interventions.^(^ ^18^ ^)^ The smallest number suggested for a two‐arm trial was 12 patients per group,^(19^ ^)^ whereas others have suggested larger numbers.^(^ ^20^ ^)^

Data were analyzed using Stata v16 (StataCorp LLC, College Station, TX, USA).

Data are presented as tables of raw data and figures showing adjusted (for age, sex, and weight) means from ANOVA models. Adjustment makes allowance for possible “confounders” that would otherwise obscure the relationships between the bone biomarkers with intervention and time; adjustment for these factors was undertaken based on consistent patterns of higher values in older, heavier girls. Any *p* values were based upon differences in adjusted means.

## 3 Results

**Fig. 1:**
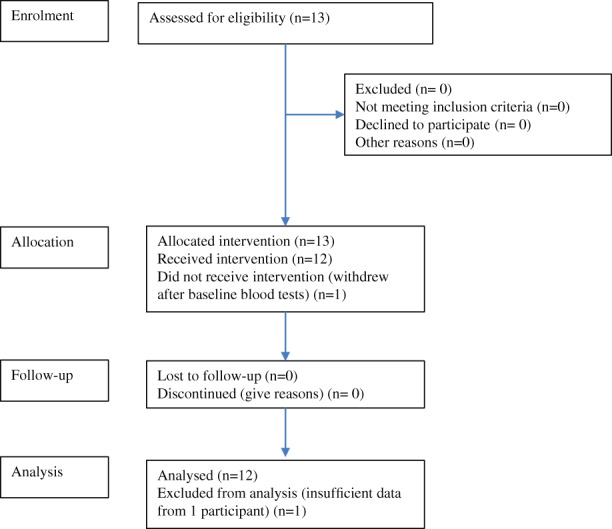
CONSORT diagram.

Thirteen children with OI, naïve to bisphosphonate treatment, were enrolled into and participated in the study between May 5, 2017, and February 8, 2018. One child withdrew after baseline blood tests were taken and before any WBV was performed; their data have been excluded. The CONSORT diagram showing the flow of patients is provided in Fig. 1.

The baseline demographics are shown in Table 1. All participants but one were either prepubertal or in early puberty, and there was a slight preponderance of girls (7/12).

**Table 1:** Study Participants' Demographic Data

ID	Age (years)	Sex	Weight (kg)	Height (cm)	Pubertal status
1	5.8	M	16.0	108.0	Stage I
2	11.3	F	36.4	140.4	Breast stage II, pubic hair stage I
3	9.9	M	26.0	137.5	Stage II
5^a^	5.6	M	21.7	101.0	Stage I
6	14.9	M	49.8	167.1	Stage IV
7	9.8	F	54.8	147.4	Breast stage II, pubic hair stage II
8	6.9	M	17.5	116 .0	Stage I
9	6.1	F	30.8	125.7	Stage I
10	5.8	F	18.6	113.0	Stage I
11	9.4	F	42.0	147.7	Breast stage II, pubic hair stage I
12	4.5	F	16.9	95.2	Stage I
14^b^	9.7	F	35.4	141.7	Breast stage II, pubic hair stage I

^a^ Number 4 withdrew.

^b^ Number 13 not assigned.

The summary unadjusted data for each biomarker at each time period are shown in Table 2. The baseline mean absolute values obtained for P1NP and CTX were around 65% of the mean values we obtained in healthy prepubertal boys^(^ ^16^ ^)^ and 80% of those in our studies of younger children;^(17^ ^)^ we did not measure BSALP in our previous studies.

**Table 2:** Raw Data Means (95% CIs) for P1NP, CTX, and BSALP

Time point (days)	Serum P1NP, ng/mL (mean, 95% CI)	Serum CTX, ng/L (mean, 95% CI)	Serum BSALP, ng/mL (mean, 95% CI)
1	464.6 (417.8, 511.5)	1.30 (1.20, 1.41)	89.8 (86.2, 93.4)
8	550.0 (499.3, 602.5)	1.48 (1.37, 1.60)	90.1 (86.3, 94.0)
15	509.5 (458.0, 561.1)	1.45 (1.34, 1.57)	90.2 (86.2, 94.2)
43	556.7 (509.8, 603.6)	1.55 (1.44, 1.66)	89.0 (85.3, 94.8)
85	460.1 (411.2, 508.9)	1.30 (1.19, 1.41)	85.9 (82.1, 89.8)
92	476.0 (424.4, 527.6)	1.42 (1.31, 1.54)	87.9 (84.0, 91.7)
99	480.5 (433.6, 527.3)	1.40 (1.30, 1.51)	88.4 (84.3, 92.3)

CI = confidence interval; P1NP = procollagen type I N‐terminal propeptide; CTX = carboxy‐terminal telopeptide of type I collagen cross‐link; BSALP = bone‐specific alkaline phosphatase.

The percentage changes in the unadjusted means from D1–8 for P1NP and CTX, were thus an increase of 18.4% and 13.8%, respectively; both markers then fell slightly (7.3% and 2.0%, respectively) from D8 to D15 and were again increased with respect to baseline at D43, 19.8% and 19.2%, respectively. The change from D43–85 across the period of risedronate therapy was effectively a return to previbration baseline levels. The percentage changes from D85–92 across the second period of vibration for P1NP and CTX, respectively, were an increase of 3.4% and 9.2%.

**Fig. 2:**
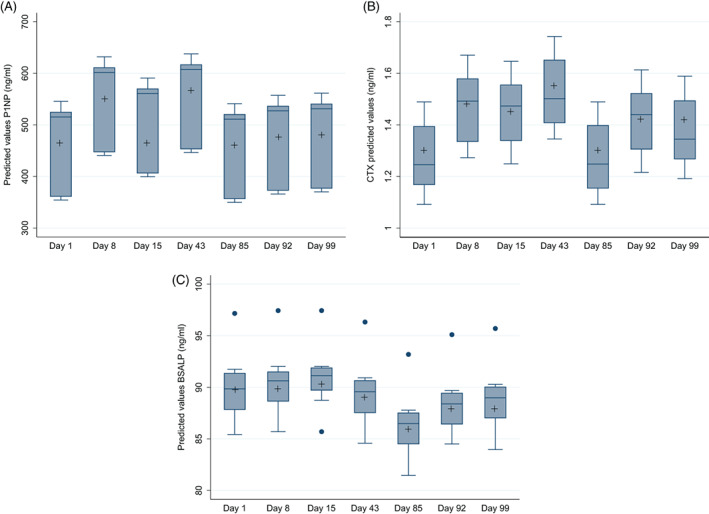
(*A*) Change in adjusted P1NP (ng/mL) across the study period. Boxes represent second and third quartiles with the internal horizontal line showing the median; whiskers are 1.5 times the interquartile range. + shows arithmetic mean; ● shows outliers more than 3 times the interquartile range from the median. (*B*) Change in adjusted CTX (ng/mL) across the study period. Boxes represent second and third quartiles with the internal horizontal line showing the median; whiskers are 1.5 times the interquartile range. + shows arithmetic mean; ● shows outliers more than 3 times the interquartile range from the median. (*C*) Change in adjusted BSALP (ng/mL) across the study period. Boxes represent second and third quartiles with the internal horizontal line showing the median; whiskers are 1.5 times the interquartile range. + shows arithmetic mean; ● shows outliers more than 3 times the interquartile range from the median.

Fig. 2*A*–*C* shows the change in each of the bone biomarkers assessed at each time point in graphical form, with the means adjusted for age, sex, and weight. For P1NP and CTX, the pattern of change is similar. For P1NP, there was a significant initial increase from D1–8 (*p* = 0.010), a plateau or slight decrement from D8–15, and then a second peak on D43 (*p* = 0.03 versus D1) before the period during which risedronate, calcium, and vitamin D were administered. For CTX, there was a significant initial increase from D1–8 (*p* = 0.014), a plateau or slight decrement from D8–15, and then a second peak on D43 (*p* < 0.001 versus D1).

After the administration of the medication between D43 and D85, P1NP fell significantly (*p* = 0.016). The change in mean adjusted CTX did not reach statistical significance (*p* = 0.086).

**Fig. 3:**
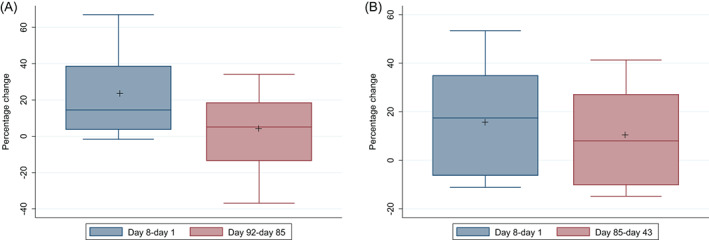
(*A*) Box plots showing comparison of P1NP percentage changes after whole body vibration period 1 (day 8–day 1) and period 2 (day 92–day 85). Boxes represent second and third quartiles with the internal horizontal line showing the median; whiskers are 1.5 times the interquartile range. + shows arithmetic mean. (*B*) Box plots showing comparison of CTX percentage changes after whole body vibration period 1 (day 8–day 1) and period 2 (day 92–day 85). Boxes represent second and third quartiles with the internal horizontal line showing the median; whiskers are 1.5 times the interquartile range. + shows arithmetic mean.

The subsequent percentage change from D85–92 in P1NP was less than it had been from D1–8 (3.7% versus 19.5%; *p* < 0.05) but the difference in percentage change in CTX (9.8% versus 14.4%) did not reach significance; Fig. 3).

The pattern of change in BSALP was different from that of P1NP and CTX. There was little change after the first period of whole body vibration from D1–42; BSALP appeared to fall from D42–85 during the period of treatment with risedronate and Calcichew and rose back toward the previbration baseline at the end of the study. The absolute changes in BSALP did not, however, reach significance at any time point (D1 versus D8 *p* = 0.822; D1 versus D43 *p* = 0.621; D43 versus D85 *p* = 0.451).

One child suffered a fractured forearm (radius and ulna) 2 days into the period of bisphosphonate administration. This was the only radiologically confirmed fracture among participants during the study.

## 4 Discussion

In 12 children with osteogenesis imperfecta, we found that the response of the bone biomarker P1NP to a standardized form of mechanical stimulation, whole body vibration, was reduced after the administration of an oral bisphosphonate, risedronate, and concomitant calcium and vitamin D supplementation. The implications of this finding are significant for the management of children with osteogenesis imperfecta, the majority of whom are likely to receive bisphosphonate treatment. Interestingly, the falls in P1NP and CTX after medication did not reduce the absolute values of either biomarker below the previbration baseline. However, the reduction in response of the serum marker of bone formation brings into question how bisphosphonates are used in children with osteogenesis imperfecta.

This is the first time that successive periods of WBV have been used to assess the effect of bisphosphonate treatment on the dynamic skeletal response to mechanical stimulation.

**Fig. 4:**
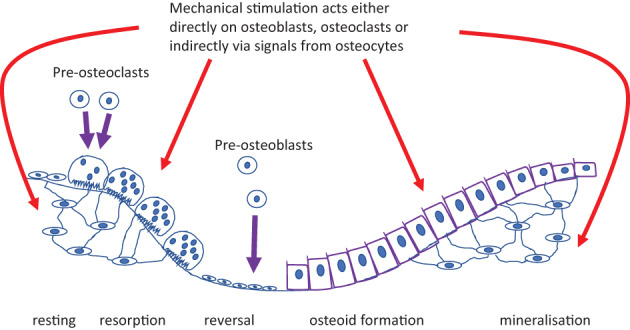
Schematic showing the possible sites of action of mechanical stimulation.

Based on our prior experience with apparently healthy children,^(16^ ^)^ we had anticipated that allowing a period of 5 weeks for the bone biomarkers to return to baseline would be adequate. We were mistaken. In contrast to the expected fall from D15 onward, we saw an increase at D43 in both P1NP and CTX compared with baseline. When we first reported on our use of this form of assessment in apparently healthy prepubertal boys, we speculated that the greater increase in P1NP as opposed to CTX was evidence of increased modeling activity in response to mechanical stimulation. On the basis of this new data, we suggest that a more plausible explanation is that WBV increases the activity of osteoblasts that are already producing bone matrix, as well as stimulating the initiation of additional remodeling events; this then is reflected in the pretreatment double peak for both P1NP and CTX; see Fig. 4 for an illustration. This action could be direct or mediated through the osteocyte network—our data are uninformative in this regard. The reduction in both P1NP and CTX after risedronate administration further reinforces the concept that cells engaged in remodeling, rather than modeling—which should be less affected by risedronate administration—are the main effectors of the response to vibration. This is, in our view, the most likely biological explanation for our findings, but we fully accept that we are only looking at circulating biomarkers of cellular activity, not at the cells themselves.

The smaller rise in P1NP in response to WBV after risedronate treatment suggests that the numbers or activity of osteoblasts forming matrix is diminished by such treatment. This would be consistent with the reports of lower osteoid surface/bone surface found during bisphosphonate treatment in children with OI.^(^ ^6^ ^)^ The fact that both CTX and P1NP did increase immediately after the second period of WBV does suggest that the capacity to respond to mechanical stimulation is still present, albeit reduced. The fact that the initial increase in CTX after WBV was not different across the two periods of vibration (D1–8; D85–92) might imply that the initiation of new remodeling events in response to vibration was similar at both times; however, the period of follow‐up after WBV2 was insufficient to demonstrate a subsequent rise in P1NP.

The pattern of change in P1NP and CTX after each period of WBV was quite different from that for BSALP. BSALP changed little in response to the first period of WBV, fell during the period of risedronate and calcium/vitamin D administration, and then rose toward the previbration baseline during and after the second period of vibration. Mineralization must necessarily come after osteoid formation; if more osteoid has been formed previously in response to WBV, it would seem logical that more mineralization would occur subsequently. It is interesting, however, to note that markers of all activities—bone resorption, osteoid formation, and mineralization—fell during the period of risedronate and calcium/vitamin D treatment. It would be interesting to speculate that this implies continuing cross‐talk between osteoclasts and osteoblasts persisting across both the period of osteoid formation and of mineralization. The change in BSALP in response to the first period of WBV is not suggestive of an immediate increase in the rate of mineralization. It is important to note, however, that none of the changes in BSALP were statistically significant.

This work has a number of limitations. The study was undertaken on a small group of children with OI and ideally the results should be confirmed in a larger group with more older children included. One child suffered a fracture during the period of bisphosphonate administration; that event may have impacted on subsequent bone marker measurements, although the individual data for that patient post‐fracture are similar to those of the other children. We did not measure factors likely to reflect osteocyte activity or osteoblast–osteoclast cross‐talk; in our previous studies, we found no acute change in circulating sclerostin or osteoprotegerin in response to WBV.^(^ ^16^ ^)^ We did not check serum vitamin D and PTH during the study; however, all children under our care with osteogenesis imperfecta are advised to take regular daily vitamin D supplements, and we also give calcium supplements to those who report low dairy product intake. The administration of calcium and vitamin D in combination with the risedronate (following the manufacturer's recommendation) may have also impacted on short‐term bone turnover. In our previous studies of children receiving risedronate in a placebo‐controlled trial,^(^ ^7^ ^)^ however, we saw no change in urinary cross‐linked N‐telopeptide of type 1 collagen at 3 months for those receiving calcium and vitamin D as placebo, in contrast to a significant decline for those also receiving risedronate. We cannot exclude the possibility that exposure to two successive episodes of WBV at this interval would result in altered bone turnover marker responses in the absence of risedronate, calcium, and vitamin D. The children in this study included girls; we saw similar percentage increases in P1NP and CTX in girls whose mothers had received supplemental vitamin D during pregnancy in the placebo‐controlled MAVIDOS study.^(^ ^17^ ^)^

In summary, we have shown that the response of bone biomarkers in children with OI to a defined period of mechanical stimulation in the form of whole body vibration is similar initially to that of normal children and significantly reduced after bisphosphonate treatment. Our interpretation of the current data set and the patterns of the response is that whole body vibration both increases the activity of osteoblasts already forming osteoid and stimulates additional bone remodeling cycles. Further work in children both with and without OI is clearly required to establish the robustness of these interpretations and determine the magnitude and duration of any such responses.

## Disclosures

NB has the following disclosures of activities within the last 5 years: principal investigator for Alexion studies of hypophosphatasia and Amgen studies of osteogenesis imperfecta; consultancy for Alexion, Amgen, Mereo, Novartis, UCB, Kyowa Kirin, and Pharmacosmos; advisory boards for Alexion, Ultragenyx, and Mereo; and speaker bureau for Alexion. All derived income passed to employers. All other authors state that they have no conflicts of interest.

### Peer Review

The peer review history for this article is available at https://publons.com/publon/10.1002/jbm4.10592.

## Data Availability

The small number of patients in this study, the rarity of the condition, and the level of detail provided in the data set risks the potential identification of individual participants. Access to the data will be through discussion with the corresponding author only.
